# The Efficacy, Safety, and Convenience of a New Device for Flushing Intravenous Catheters (Baro Flush™): A Prospective Study

**DOI:** 10.3390/medicina56080393

**Published:** 2020-08-05

**Authors:** Youn I. Choi, Jae Hee Cho, Jun-Won Chung, Kyoung Oh Kim, Kwang An Kwon, Han Yong Chun, Dong Kyun Park, Yoon Jae Kim

**Affiliations:** 1 Department of Gastroenterology, Gil Medical Center, Gachon University, Incheon 21565, Korea; cys7like@hanmail.net (Y.I.C.); jhcho9328@gachon.ac.kr (J.H.C.); drgreen@gilhospital.com (J.-W.C.); kkoimge@gilhospital.com (K.O.K.); toptom@gilhospital.com (K.A.K.); pdk66@gilhospital.com (D.K.P.); 2 Center for Medical Robotics, Korea Institute of Science and Technology, Incheon 21565, Korea; Hychun75@kist.re.kr

**Keywords:** flushing, venous catheter, occlusion, catheter infection

## Abstract

*Background and Objectives*: An effective flushing technique is essential to reduce intravenous (IV)-related complications and improve patient care. New technology should contribute to such improvements, while reducing costs and increasing care efficiency. This study evaluated the efficacy, safety, and convenience of a new flushing technique using a Baro Flush™ controller. *Materials and Methods*: We evaluated the efficacy and safety of Baro Flush™ by measuring the infusion flushing volume and pressure in vitro. Afterwards, we prospectively enrolled 3000 patients with flushing and assigned 1500 patients with a new technique for flushing and 1500 with a conventional flushing method, which was performed by 48 registered nurses (RNs) at the Gil Medical Center in June 2018. The efficacy, safety, and convenience of the new flushing method were evaluated though a questionnaire survey. *Results*: The average flushing pressure was 12.5 ± 0.6 psi (86.18 ± 4.14 kPa) with 1.2 ± 0.2 mL per flush, as recommended by the Centers for Disease Control and Prevention based on 85 experiments. No IV-catheter-related complications were reported by the RNs during the study. More than 80% of the RNs reported that the new flushing method was easier to learn, improved care efficacy, and was more convenient than conventional flushing. *Conclusions*: The new flushing method using a Baro Flush™ controller showed improved efficacy, safety, and convenience compared with the conventional flushing method, and no IV-catheter-related complications occurred, including occlusion and inflammation. The new flushing method promises to reduce IV-catheter-related complications and shows improved efficacy, safety, and convenience.

## 1. Introduction

Venous cannulation via a central or peripheral intravenous (IV) catheter is one of the most frequently performed health care procedures, with up to 80% of all hospitalized patients requiring medication, nutrition, or transfusion through IV catheters [1,2,3]. Although IV catheter insertion, and maintenance are common procedures, studies have reported that IV-catheter-related complication rates range from 2% to 80%, where complications include phlebitis (inflammation of the vein wall), occlusions (blockage), infiltration/extravasation (fluid moving into the third compartment), and, rarely, infections [2,4,5,6].

According to the Centers for Disease Control and Prevention (CDC), it is important to flush the catheter under positive pressure to reduce IV-catheter-related complications, maintain patency, and prevent the mixing of incompatible medications [4,7,8,9]. Although there is no consensus on the optimal frequency of catheter flushing [7], the Royal College of Nursing recommends flushing more than two times a day [10] to prevent peripheral IV-catheter-related complications [4,9,11,12]. Because each flushing procedure has to be done using aseptic techniques, IV-catheter flushing is a burden on registered nurses (RNs) and is also time consuming and expensive [11,13,14].

Conventional flushing methods usually involve several steps: preparing the flushing fluid (sterile 0.9% sodium chloride) in a 10 mL syringe; cleaning the access port with a single-use 70% alcohol-impregnated swab or 2% alcohol chlorhexidine thoroughly for at least 15 s, and allowing it to dry before accessing the system [7,10,15]. Often, physicians and RNs find it hard to follow these recommendations, since IV-catheter flushing techniques should be done with aseptic and complex procedures [13]. According to a recent report that examined the real-world practice of IV-catheter insertion and maintenance, more than 15% of clinicians did not follow the recommendations [16,17,18]. Therefore, simple, convenient, infection-free techniques are needed.

Flushing with an aseptic non-touch technique using a prefilled syringe was introduced as an alternative to conventional methods; although it eliminated preparation of the flushing fluid, it still needed several complex steps [19]. Positive- or negative-pressure mechanical valves with needleless connectors were also introduced. However, owing to the increased rates of IV-catheter-related infection, the CDC recommended against physicians using these methods [20,21].

Therefore, we evaluated the efficacy, convenience, and safety of the new Baro Flush™ flushing method in a prospective study population.

## 2. Methods

### 2.1. Characteristics of the New Flushing Method Using the Baro Flush™ Controller

**Figure 1:**
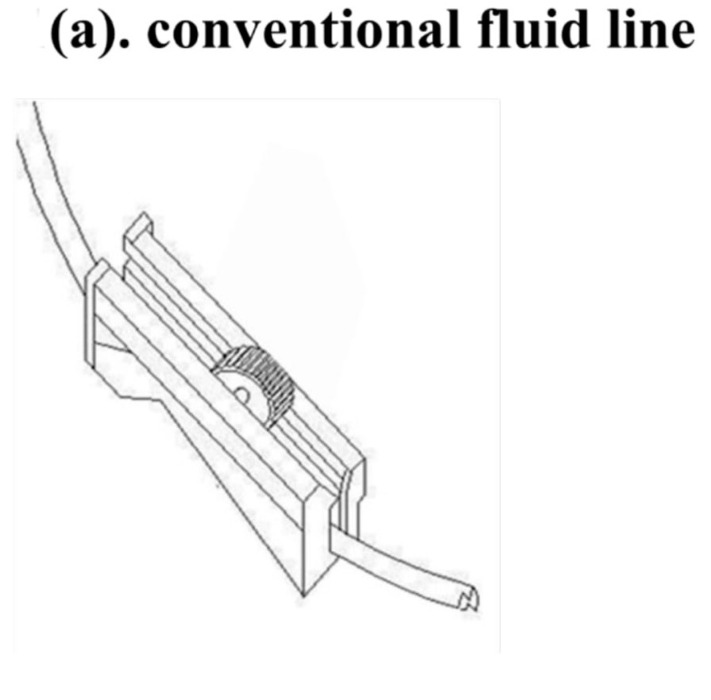

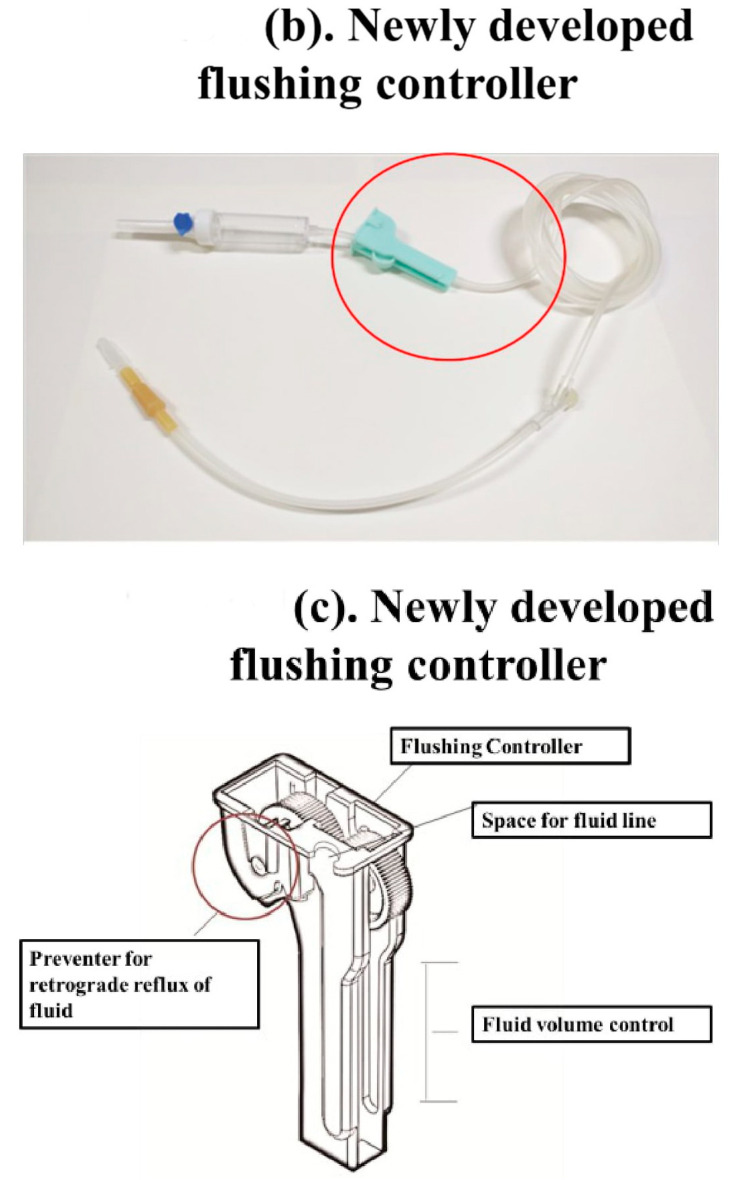

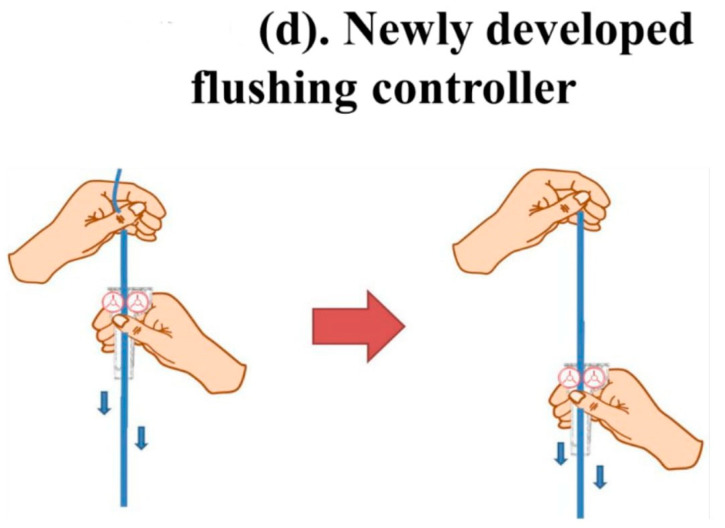
The new Baro Flush™ flushing controller. (**a**) Conventional fluid line. (**b**) The Baro Flush™ controller. (**c**) Illustration of Baro Flush™, and (**d**) operation of Baro Flush™. The externally located Baro Flush™ flushing controller was modified from a conventional fluid volume controller and has the added function of flushing. To operate Baro Flush™, the controller is rolled down and up the intravenous (IV) line twice.

The new flushing set is a modification of a conventional IV set, especially the fluid volume controller. To manipulate the flushing controller, the flushing controller is pulled along a conventional fluid line and down and up twice to generate sufficient flushing volume and pressure (Figure 1).

To evaluate efficacy, convenience, and safety of the new Baro Flush™, we conducted a study in two steps. In the first step, we conducted an in vitro study to find the proper flushing volume and pressure before applying it directly into patients. In the second step, we prospectively enrolled 3000 patients who were admitted into the Gil Medical Center (GMC) and assigned into 1500 patients with the conventional flushing method and 1500 patients with the new flushing technique.

### 2.2. Efficacy and Safety of the New Flushing Method Using the Controller

**Figure 2:**
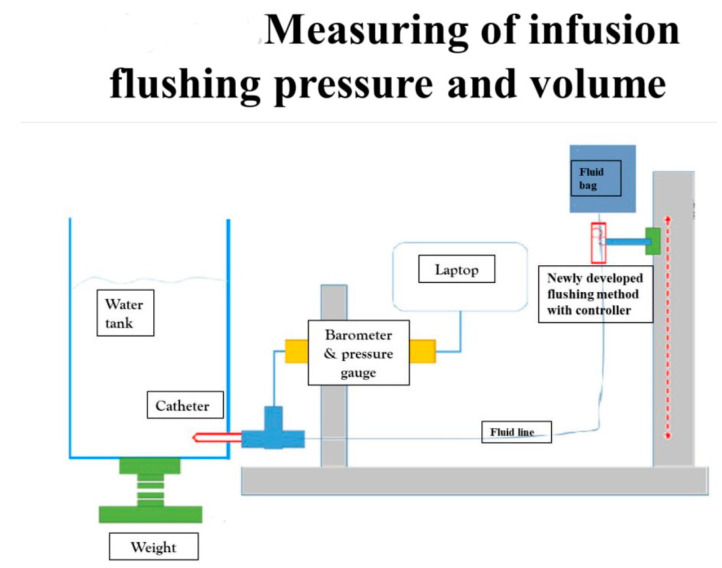
Measuring the infusion flushing pressure and volume. To measure the flushing pressure with our new flushing method, we used (1) a water tank, (2) fluid bag, (3) barometer, and (4) connecting fluid lines. Using our new flushing controller, we measured the flushing volume and pressure on the water tank with the pressure gauge.

We evaluated the efficacy and safety of the new Baro Flush™ flushing method by measuring the infusion flushing pressure and volume in vitro. CDC guidelines recommend an infusion pressure of less than 25 psi and a minimum flushing volume of 2 mL for one flush [7,8,22]. To measure the flushing pressure using Baro Flush™, we connected a barometer directly to the system. To measure the flushing volume, we devised the experiment shown in Figure 2. The experiment involved (1) a water tank, (2) a fluid bag supplying water to the water tank, (3) a pressure gauge, and (4) connecting fluid lines. We weighed the water in the tank to calculate the flushing volume.

We repeated this experiment 85 times by having 13 RNs and four physicians perform the experiment five times each.

### 2.3. Assessing the Learnability, Effectiveness, and Convenience of the New Method

We prospectively enrolled 48 RNs at the GMC who performed 3000 cases of IV-catheter flushes in June 2018: 1500 cases (1500 patients) using the Baro Flush™ and 1500 cases (1500 patients) using the conventional method.

We prospectively enrolled 3000 consecutive patients who were admitted into the GMC during the study period, who agreed to be enrolled in this study, and who signed into written consent.

The inclusion criteria for registered nurses is as below: First, they had to agree to participate in this study and sign written informed consent. Second, they had to work as registered nurses in the ward in the GMC. Third, they had to have experience using flushing sets for patients. Fourth, they had to have experience flushing for patients. The inclusion criteria for enrolled patients was as follows: they were supposed to have an intravenous catheter more than 48 h after admission.

Exclusion criteria for enrolled patients and registered nurses were as follows: first, those who refused to participate in this study; second, those who were not supposed to have an intravenous catheter in less than 48 h. Since Baro Flush^TM^ was made for any patient who uses an intravenous catheter, we did not set any other exclusion criteria for this study except for the aforementioned one to avoid allocation bias.

Using a questionnaire, the 48 RNs evaluated the efficacy, convenience, and safety of the new, and conventional flushing methods. The questionnaire included four items: (1) learnability, (2) efficacy, (3) convenience, and (4) safety. Complications of the new flushing technique using the controller were closely monitored by qualified RNs.

We monitored patients with flushing-related complications during the admission period and more than one week after discharge.

### 2.4. Statistical Analysis

The surveys consisted of close-ended multiple-choice questions and hybrid questions (i.e., rate the importance or provide numbers), which are summarized descriptively in proportion with their 95% confidence intervals. The nonparametric Mann–Whitney U test was used for the analysis. Categorical variables were reported as a percentage and analyzed using the chi-square or Fisher’s exact test. A *p*-value ≤ 0.05 was deemed to be significant. All statistical analysis was performed using SPSS software (ver. 20.0; SPSS Inc., Chicago, IL, USA).

### 2.5. Ethics

The study design, survey protocol, and related documentation were reviewed and approved by the GMC Research Ethics Board (GMC IRB number: GBIRB2017-124).

## 3. Result

### 3.1. Efficacy and Safety of the New Flushing Method Using the Baro Flush™ Controller

Using Baro Flush™, the average ± standard deviation of flushing infusion pressure was 12.5 ± 0.6 psi, (86.18 ± 4.14 kPa), and the mean flushing volume was 1.2 ± 0.2 mL per flush based on 85 experiments.

No case of occlusion or phlebitis was reported in either group during the study period.

### 3.2. The Learnability, Effectiveness, and Convenience of the New Method Assessed by RNs

Regarding the convenience of Baro Flush™, 70.0% (n = 33) of the participants indicated that the new method had good to excellent convenience compared with the conventional method (Figure S1a,b, Table 1 and Table 2). Regarding learnability, 100% (n = 48) of the participants indicated that the new method was easier to learn and use than the conventional flushing method (Figure S1a,b, Table 2). Moreover, more than 95% of the participants indicated that the new method improved the quality, safety, and speed of flushing.

## 4. Discussion

This study evaluated a new flushing method using the Baro Flush™ controller without the need for flushing syringes and evaluated its efficacy, convenience, and safety compared with the conventional flushing technique. The new flushing method reduced the risk of IV-catheter-related irritation and infection compared with the conventional method. Moreover, the learnability, efficacy, and convenience of the new method were higher than for the conventional method (*p* < 0.01). Therefore, this method is a promising alternative to the conventional flushing method.

There were several differences between our newly developed flushing device and the conventional IV infusion pump system. First, our newly developed flushing device is to be used for effective flushing not to be used for continuous drug infusion. While the conventional device, the IV infusion pump, is generally used for continuous drug infusion or transfusion, our newly developed device [23,24], the Baro Flush™, is to be used for effective flushing with proper pressure and volume. Baro Flush™ is made for effective and convenient flushing in a simple way, especially when patients have to keep their intravenous catheter for long-term infusion with proper scheduled flushing. Second, the conventional IV infusion pump is so expensive that it is impossible to apply an IV infusion pump to all patients. However, the Baro Flush™ is a simple but effective device. In this regard, our new device is somewhat different from automated IV infusion pumps.

There have been several attempts to overcome the pitfalls of conventional flushing methods. Flushing with prefilled syringes and positive- or negative-pressure mechanical valve needleless connectors has been introduced in clinical practice. When using prefilled syringes, RNs and clinicians do not need to prepare the flushing syringes [19]. Otherwise, there is no difference from the conventional method. Moreover, considering cost-effectiveness, flushing with a prefilled syringe is expensive [19]. A recent report on real-world IV-catheter management found that RNs and clinicians do not follow the recommendation “one needle, one syringe, and only one time” [16]. Moreover, a recent observational study found that only 25% of RNs performed handwashing or gloving before or after an injection [13]. Therefore, more convenient aseptic techniques for real-world situations are needed. Another alternative method, i.e., the use of positive- or negative-pressure mechanical valve needleless connectors, has been withdrawn due to an increased risk of bloodstream infections.

As an alternative, we evaluated an external flushing controller, the Baro Flush™, which is a modified version of the conventional fluid volume controller with the added function of flushing. The Baro Flush™ procedure consists of pulling the controller down and up along the IV line twice. Consequently, no aseptic procedures are needed for managing the controller, and no flushing syringes or accessing ports are required. This simplified technique is easy to learn and use and is effective. RNs reported that the new method was superior to the conventional method in terms of learnability, efficacy, convenience, and safety.

The average flushing pressure using Baro Flush™ in 85 experiments was 12.5 psi with 1.2 mL per flush. According to CDC guidelines [7], the infusion pressure should never exceed 25 psi to prevent damage to blood vessels, and a minimum flushing volume of 2 mL of flushing solution is considered to be sufficient. Therefore, the CDC guidelines recommend using a 10-mL syringe with 2 mL of flushing fluid for conventional flushing. Because the Baro Flush™ method provides the recommended flushing pressure and volume, it is safe for use. During our study, there were no complications, such as peripheral IV-catheter occlusion, inflammation, or thrombophlebitis using the new flushing method in 1500 cases. Our flushing method is simple and easy to learn compared with conventional flushing methods, which should reduce the error rate. In addition, our flushing method does not require the use of gloves, syringes, or other costly items.

Our study had several limitations. Although 3000 cases of flushing were examined, the study was conducted in a single center. Therefore, a larger study population in a multicenter randomized controlled trial is needed. Second, we monitored complications only for up to 7 days. However, IV-catheter-related complications usually occur within 7 days [7].

## 5. Conclusions

In conclusion, the new flushing method using a flushing controller is a promising alternative to the conventional flushing method in terms of efficacy, safety, and convenience of use to prevent IV-catheter-related complications.

## Figures and Tables

**Table 1:** Questionnaire survey used to assess the learnability, effectiveness, and convenience of the new Baro Flush™ method used by registered nurses (RNs).

Questionnaire	Overall Satisfaction		*p*-Value
	Good to Excellent	Fair or Poor	
**Learnability**			
Easy to learn how Baro Flush™ works	45 (93.8%)	3 (6.3%)	0.001
**Efficacy**			
Effective flushing	48 (100.0%)	0 (0.0%)	<0.001
**Convenience**			
Flushing performance velocity	43 (89.6%)	5 (10.4%)	0.005
Easy to manipulate	46 (95.8%)	2 (4.2%)	0.001
**Safety**			
Risk of needle stick injury	48 (100.0%)	0 (0.0%)	<0.001

**Table 2:** Questionnaire comparing Baro Flush™ with the conventional flushing method used by RNs (n = 48).

Questionnaire Variables	Overall Satisfaction with Baro Flush™ Compared with the Conventional Method		*p*-Value
	Good to Excellent	Fair or Poor	
**Learnability**	48 (100%)	0 (0%)	<0.001
**Efficacy**	48 (100%)	0 (0%)	<0.001
**Convenience**	48 (100%)	0 (0%)	<0.001
**Safety**	48 (100%)	0 (0%)	<0.001

## Supplementary Materials

The following are available online at https://www.mdpi.com/1010-660X/56/8/393/s1, Figure S1: Questionnaire survey for assessing the learnability, effectiveness, and convenience of the new Baro Flush™ method by registered nurses (RNs).
