# Supplementary material for: The Efficacy, Safety, and Convenience of a New Device for Flushing Intravenous Catheters (Baro Flush™): A Prospective Study
Source: Medicina (Kaunas). 2020 Aug 5;56(8):393. doi: 10.3390/medicina56080393 (PMC7466298; doi:10.3390/medicina56080393)
Supplement: Supplementary file 1 [file medicina-56-00393-s001.pdf]

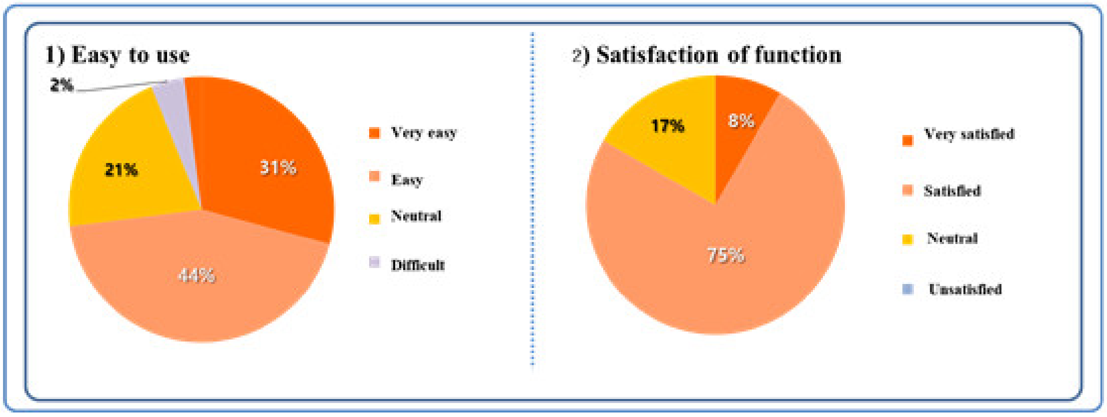

a

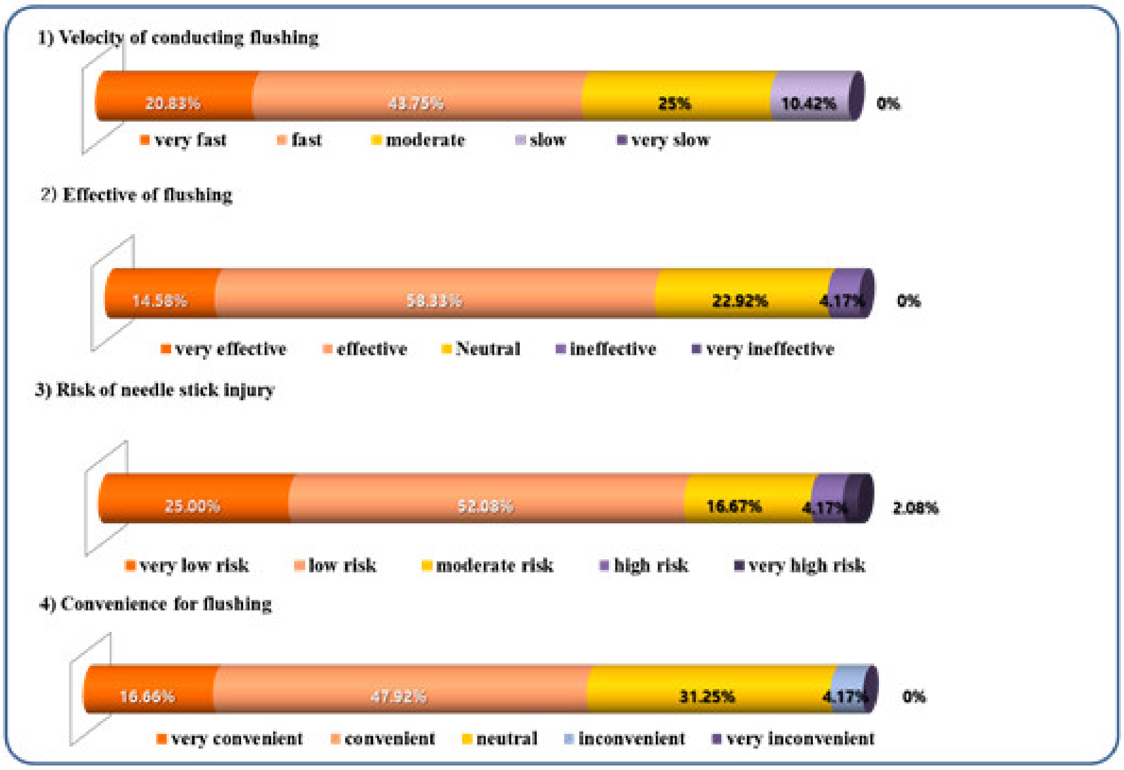

b

Figure S1. Questionnaire survey for assessing the learnability, effectiveness, and convenience of the new Baro Flush™ method by registered nurses (RNs). (**a**) Results for Questionnaire comparing Baro Flush™ with the conventional flushing method used by RNs, (**b**) Assessing the learnability, effectiveness, and convenience of the new Baro Flush™ method.
